# Modeling Arabidopsis root growth and development

**DOI:** 10.1093/plphys/kiaf045

**Published:** 2025-02-27

**Authors:** Marta Ibañes

**Affiliations:** Departament de Física de la Matèria Condensada, Universitat de Barcelona, Martí i Franquès 1, 08028 Barcelona, Spain; Universitat de Barcelona Institute of Complex Systems (UBICS), Universitat de Barcelona, 08028 Barcelona, Spain

## Abstract

Modeling has been used to explore various aspects of primary root development and growth in *Arabidopsis thaliana*, thanks to enormous advances in the genetic and biochemical bases of cell division, cell growth and differentiation, and, more recently, progress in measuring these processes. Modeling has facilitated the characterization of the regulations involved in these processes and the system properties that they confer. Recently, the mechanical-physical properties of root growth have started to be determined with the help of modeling. Here we review recent progress in modeling approaches used to examine root development and growth, from the transcriptional and signaling regulation of cell decisions to the mechanical basis of morphogenesis, and we highlight common features and future challenges.

## Introduction

Curiosity and the desire to understand, control, and shape our environment are unique traits of humankind. Among the many subjects we seek to explore and manipulate, living matter stands out, and we have made extraordinary advances in this field over the past decades. These advances have been significantly aided by computational and mathematical modeling of living systems. Modeling serves as a benchmark to test the plausibility of our understanding of a phenomenon and to challenge it with specific predictions. Through modeling, we can assess whether known interactions and players are sufficient and necessary to account for a phenomenon. Modeling allows us to explore the implications of our assumptions and hypotheses, and, by comparing the consequences with observed reality, we gain a deeper knowledge and understanding (Lander 2014).

When modeling a system, the first step is the most crucial and complex, while subsequent ones are more straightforward (Strogatz 2019). In this initial step, the modelers must determine which factors are relevant and which can be disregarded—a decision that depends on the specific question being addressed, what we aim to learn, and the desired level of abstraction. The subsequent steps formalize the results of this first step through mathematical equations and/or computational rules. It is worth emphasizing that the theoretical formulation of the model enables us to make use of mathematical derivations and to unravel relationships between phenomena that were not initially evident. Model development that requires empirical data involves 2 additional and highly relevant steps, namely the interpretation of the empirical data and its relationship with the model outcomes. Failure in either of these steps can lead to meaningless results and may even cause confusion. Therefore, modeling must always be approached with care.

This Review focuses on recent biochemical and biophysical models of primary root development and growth in the model organism *Arabidopsis thaliana* (Arabidopsis). It covers a range of topics, from the transcriptional and signaling regulation of cell decisions to the mechanical basis of morphogenesis, exemplifying how we have gained new understanding of the phenomenon under study through modeling. Despite space limitations that impede detailing the technological and biological advances pivotal for the establishment of these models, the synergy provided by the combination of modeling with experiments is highlighted when possible.

### Modeling TF dynamics at the root stem cell niche

The tip of the primary root of Arabidopsis has a stereotyped geometry that is conserved during root growth through a regulated pattern of cell divisions (Dolan et al. 1993). The root structure comprises longitudinal layers—each with its own properties and characteristics—that originate from the stem cell niche, located close to the tip. At the center of the niche, there is a set of a few cells that rarely divide and that form the quiescent center (QC). Surrounding the QC, there are the stem cells whose divisions give rise to the different cell layers. Divisions can be asymmetrical (also named formative), in which case the division event gives rise to 2 cells that are positioned in separate layers. Divisions can also be symmetrical (also named proliferative), in which case the 2 newborn cells are equivalent and contribute to the same cell layer. Research in recent decades has detailed this anatomical structure, the planes of cell divisions, and a myriad of genes that regulate these cell divisions.

**Figure 1.**
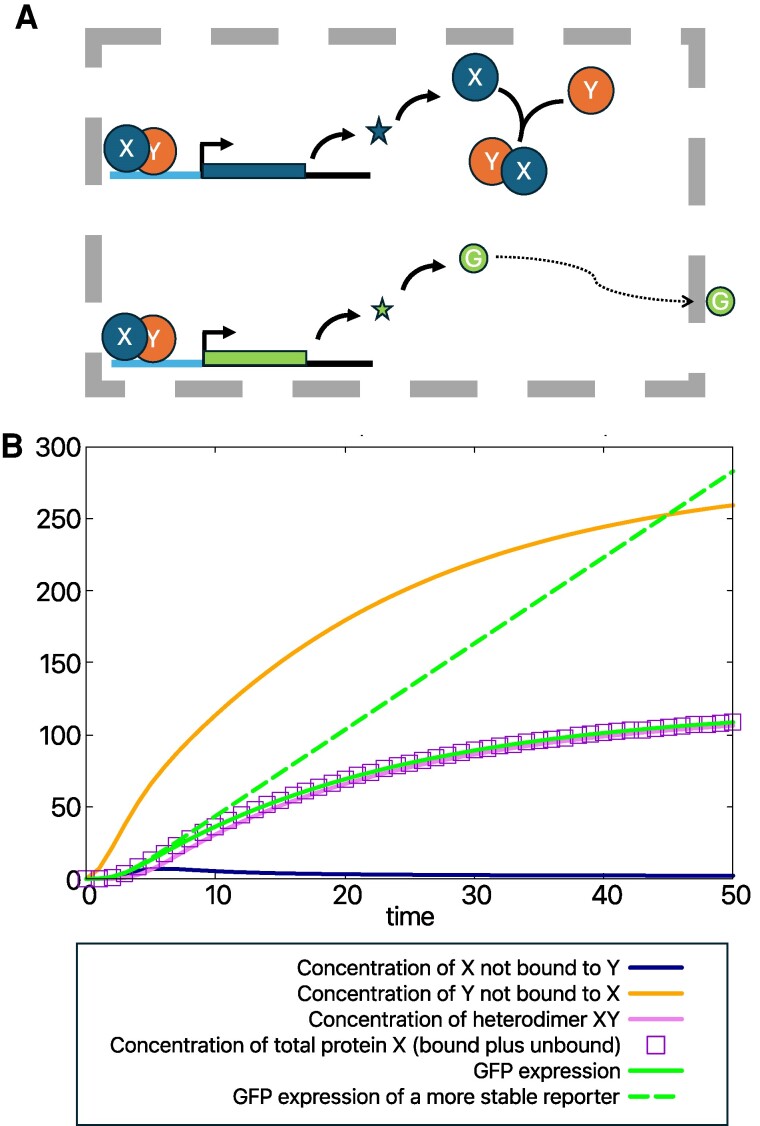
Modeling heterodimer formation and promoter expression through the GFP reporter. **A)** Cartoon depicting transcription of mRNA *x* (star) and translation to protein X (circle). Heterodimer formation with protein Y is depicted without arrows. The promoter is depicted with a light blue line. Modeling of GFP expression of the promoter of gene X is also shown. The GFP protein can diffuse to the outside of the cell (delimited by dashed grey lines). The promoter is activated by the heterodimer. **B)** Temporal evolution of the regulations in A when all transcripts and proteins are initially absent, and Y is transcribed constantly. GFP is assumed to have the same half-life and transcriptional and translation rates as species X and not to diffuse. In this case, GFP is a good dynamical reporter of the total concentration of protein X (bound plus unbound to Y). Notice that most X is bound to Y. A GFP reporter with a longer half-life (stable GFP) is not an appropriate dynamical reporter. Modeling is done as explained in Boxes 1 and 2 and the plot depicts the concentrations of molecules over time.

The formation of heterodimers of transcription factors (TFs) is a common phenomenon involved in the regulation of cell divisions in the stem cell niche. For instance, the TFs SHORT ROOT (SHR) and SCARECROW (SCR) form heterodimers with distinct stoichiometries (Clark et al. 2020), and they drive asymmetric cell division of the cortex/endodermis initial, giving rise to a cortex cell and an endodermal cell (Cruz-Ramírez et al. 2012). In contrast, symmetric divisions of this initial do not require the heterodimer. Another example is provided by the TFs BRASSINOSTEROIDS AT VASCULAR AND ORGANIZING CENTER (BRAVO) and WUSCHEL-RELATED HOMEOBOX 5 (WOX5), which bind together and repress cell divisions of quiescent center cells (Betegón-Putze et al. 2021). This repression is lifted when the phosphorylated form of a TF that mediates Brassinosteroid hormone signaling (BES1) binds to BRAVO (Vilarrasa-Blasi et al. 2014). An additional common aspect in the regulation of cell divisions in the stem cell niche is that one of the TFs forming the heterodimer transcriptionally regulates the other, thereby creating a feedback loop (Fig. 1A). In this regard, SHR induces SCR (Levesque et al. 2006; Cui et al. 2007), and BRAVO is induced by WOX5 (González-García et al. 2011; Betegón-Putze et al. 2021) and repressed by BES1 (Vilarrasa-Blasi et al. 2014).

Modeling has proved helpful to identify these interactions. Models of the interactions between TFs can be formulated, and the predicted TF concentrations can be compared with measurements of expression data. These models can be expressed in terms of ordinary differential equations, as described in Box 1. Furthermore, the relationship between the modeling outputs and fluorescent reporters can be defined as detailed in Box 2. By comparing the model outputs to experimental measurements under different perturbations or at different time points, it is possible to propose which regulations are most plausible (Clark et al. 2019, 2020; Betegón-Putze et al. 2021).

Box 1. **Modeling gene regulation**

The dynamics of gene-protein regulatory interactions in a cell can be modeled through ordinary differential equations (ODEs). These establish the rates of change of the average concentrations of the transcripts and proteins being modeled. An example is given below, where 2 genes, X and Y, are considered and the dynamics of their transcripts and proteins are modeled through the following ODEs:

$$\frac{dx}{dt}=P_{x}(X,Y,H)-d_{x}x$$

$$\frac{dy}{dt}=P_{y}(X,Y,H)-d_{y}y$$

$$\frac{dX}{dt}=r_{X}x-d_{X}X-k_{b}X\cdot Y$$

$$\frac{dY}{dt}=r_{Y}y-d_{Y}Y-k_{b}X\cdot Y$$

$$\frac{dH}{dt}=k_{b}X\cdot Y-d_{H}H$$

In these equations, *t* denotes time, *x* and *y* stand for the concentrations of each transcript, and *X*, *Y*, and *H* are the concentrations of the proteins when not bound together (*X*, *Y*) and when bound (i.e. the concentration of heterodimer, *H*). Translation rates per concentration unit of transcript are *r_X_*, *r_Y_* for genes X and Y, respectively. The transcription rates of genes *X* and *Y* are $\;P_{x}(X,Y,H)$ and $P_{y}(X,Y,H)$, respectively. These rates depend on genes *X* and *Y* through their proteins in their unbound forms and their heterodimer forms. This dependence can be settled through reaction-schemes and mass-action law or phenomenologically.

The formation of heterodimers involves the conversion of unbound molecules into heterodimers. Using mass-action law, this is modeled by the velocity of this formation as $k_{b}X\cdot Y$, which contributes negatively to the rate of change of *X*, *Y* and positively to the rate of change of *H*. The last term in all equations stands for the destruction or removal of the corresponding transcript or protein (assumed to take place at a constant rate per unit of concentration). The heterodimer is eliminated and therefore does not accumulate indefinitely. In the above model, heterodimer dissociation has been omitted. However, we could instead have formulated the model with heterodimer dissociation and without heterodimer elimination. These decisions, which need to be taken when establishing a model, can be based on data. When data are absent, the relevance of these assumptions can be studied to determine whether these are crucial for the conclusions drawn from the model. Alternatively, other formalisms that require less parameters, like Boolean descriptions, can be used.

Box 2. **Gene regulation and expression data**

The transcription functions $P_{x,y}(X,Y,H)$ can be phenomenological or formulated through mass-action law and are constructed based on data about how genes X and Y affect their expression.

**Figure 2.**
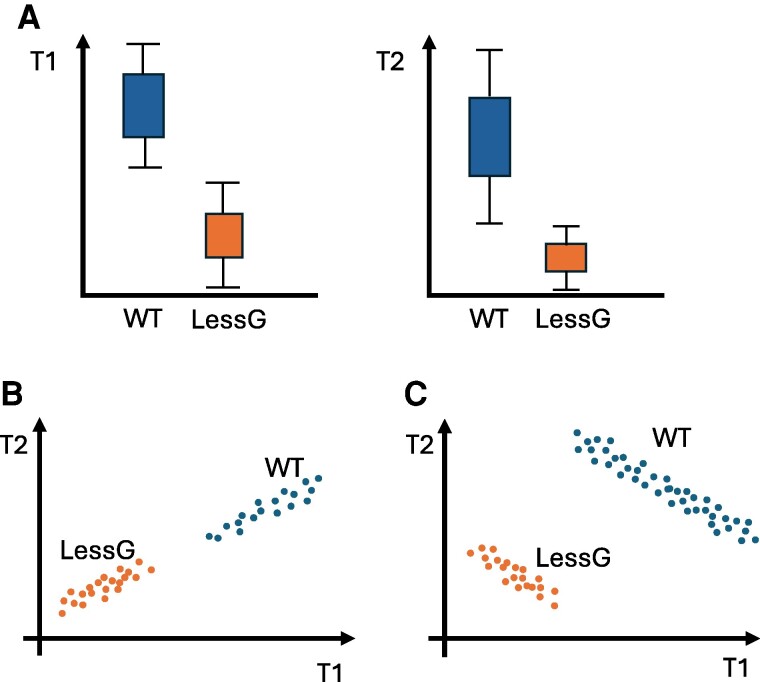
Correlations provide information. The figure shows invented data. **A)** Cartoon of boxplots for 2 morphological traits (T1 and T2) in the wild type (WT) and in a perturbed case where factor G is altered compared with the WT (LessG, e.g. gene G is knocked down). **B)** When T1 and T2 are measured in the same sample, the (T1, T2) values of each sample can be represented in a 2D plot. Each circle denotes the values for 1 sample. In B, the data from the samples show a positive linear correlation. These data are the same as those depicted in A. **C)** Alternative scenario to B. The data from A, when plotted in the 2D T1-T2 plane could be like the plot shown in C. In this case, the WT and the perturbed case show a negative linear dependence of T1 and T2, which is not explained by G. Therefore, the 2D plots, B and C, are compatible with A and provide additional information that can be used to construct models or to validate the model predictions.

When gene expression of X is visualized through GFP of the promoter of gene X, the dynamics of GFP can be modeled as (Mercadal et al. 2022) (Fig. 2B)

$$\frac{dg}{dt}=P_{x}(X,Y,H)-d_{gfp}g$$

$$\frac{dG}{dt}=r_{g}g-d_{GFP}G,$$

where *G* is the GFP concentration (hereafter named GFP expression) and *g* is the concentration of its transcript. Therefore, in the stationary state $\left(\frac{dg}{dt}=0,\;\frac{dG}{dt}=0\right)$, GFP expression is proportional to the promoter rate, being

$$G=\frac{r_{g}}{d_{gfp}d_{GFP}}P_{x}(X,Y,H)$$

and *X*, *Y*, *H* are obtained from their rate of change also established at the stationary state (i.e. for $\frac{dx}{dt}=0,\;\frac{dX}{dt}=0,\frac{dy}{dt}=0,\frac{dY}{dt}=0,\frac{dH}{dt}=0$).

This approach enables us to model GFP expression of gene X when gene Y is absent (for instance, if protein Y is knocked down) as

$$\frac{dg}{dt}=P_{x}(X,Y=0,H=0)-d_{gfp}g$$

$$\frac{dG}{dt}=r_{g}g-d_{GFP}G,$$

and *X* is obtained from its rates of change $\left(\frac{dx}{dt},\;\frac{dX}{dt}\right)$ when *Y* = 0 and *H* = 0.

The models in Boxes 1 and 2 assume no spatial dependence, yet the transport of molecules and spatial regulation of the synthesis and elimination of molecules can be included to address spatial patterning.

In vivo time-lapse imaging of proteins is done through fluorescently tagged proteins. If the endogenous protein is removed and substituted by a fluorescent one, which functionally behaves like the endogenous one (Winter et al. 2024), then the dynamics of this fluorescent reporter can be modeled as (using the dynamics in Box 1)

$$\frac{df}{dt}=P_{y}(X,F,HF)-d_{f}f$$

$$\frac{dF}{dt}=r_{Y}f-d_{F}F-k_{b}X\cdot F$$

$$\frac{dHF}{dt}=k_{b}X\cdot F-d_{HF}HF,$$

where *f* stands for the concentration of transcript, *F* for the concentration of fluorescent protein when it is not bound to X, and *HF* for the concentration of the heterodimer formed by X with the fluorescent protein. The dynamics of gene X (rate equations for *x* and *X*) proceed as in Box 1 but substituting *Y* by *F* and *H* by *HF*. The reporter measures both the unbound and bound form of the fluorescent protein and therefore corresponds to F(t)+HF(t).

Modeling has also proved useful to unravel the specific properties that result from these regulations. For instance, modeling the binding and unbinding dynamics of TFs to form heterodimers has revealed that heterodimer formation confers ultrasensitivity to changes in TF concentrations (Buchler and Louis 2008). To simplify, the fraction of one of the TFs in the bound form (i.e. in heterodimer form) relative to its total amount (both bound and unbound) is either close to 0 or close to 1, depending on the concentration of the other TF. By adding the transcriptional regulation of one TF by the other in models of heterodimer formation at the root stem cell niche, we have learned that this feedback enhances ultrasensitivity. Since hormones regulate the concentrations of these TFs in the root stem cell niche, these TFs respond to hormonal changes not in a graded manner, but with ultrasensitivity. For instance, BRAVO is repressed with ultrasensitivity by brassinosteroid signaling, allowing the QC to divide once the brassinosteroid signaling surpasses a certain threshold (Vilarrasa-Blasi et al. 2014), and the binary response of SHR and SCR to the gradient of phytohormone auxin restricts the heterodimer SHR-SCR to the cortex/endodermis initials (Cruz-Ramírez et al. 2012).

Recently, time-lapse microscopy has been used to visualize SCR and SHR dynamics in vivo within the nuclei of endodermal cells when being induced in *shr2* mutants (Winter et al. 2024). The dynamics of the SHR and SCR reporters are consistent with an ultrasensitive behavior (Winter et al. 2024) but not with expected bistability of the heterodimer (Cruz-Ramírez et al. 2012). Future studies are therefore required to address these dynamical data in the context of heterodimer formation. In addition, these reporter dynamics have been analyzed with machine learning techniques to identify the features that predictively discriminate symmetric from asymmetric cell divisions. Based on this analysis, it has been proposed that the concentration of SCR or SHR in a cell only during the early phase of the cell cycle indicates whether the cell will divide symmetrically or asymmetrically. This finding highlights an additional layer of regulation: the window of the cell cycle during which cells are competent to receive information on how to proceed.

A further common phenomenon involved in the regulation of cell divisions and differentiation at the stem cell niche is that TFs can move between cells, such that they can act beyond their domain of expression. The movement of these TFs is short range, involving few cells. This is the case of SHR, which moves from inner tissues to the endodermis, where it regulates the type of cell division (Helariutta et al. 2000). It is also the case of WOX5, which moves from the QC toward the columella and to the vascular initials (Pi et al. 2015; Berckmans et al. 2020; Clark et al. 2020). Modeling has shown that the regulatory interactions between the TFs can confine their expression to a specific domain of cells rather than a broader area. This happens when a mobile TF activates another TF that sequesters it, through binding, and impedes its movement, as proposed to occur between the mobile WOX5 and immobile BRAVO at the stem cell niche (Mercadal et al. 2022).

Does the movement of TFs confer additional properties? If SCR is required in the endodermis to regulate symmetric vs asymmetric cell divisions, why is it not produced directly by endodermal cells, instead of reaching these cells through movement? In the future, formulating models can help us reach conclusions about the trade-offs that influence the movement of these molecules compared with other mechanisms.

### The long-range transport of auxin

Plants have several long-range transport mechanisms that enable communication between distant parts, like the shoot and the root. One of these long-range transport systems is that of the phytohormone auxin. Auxin regulates many processes in plant development and is relevant for the spatial organization of the root. Auxin concentration and signaling are not homogeneously distributed along the root but peak at the QC. Over the last 2 decades, multiple modeling studies have been undertaken to explore how the nonhomogeneous distribution of auxin is established in the primary root (Swarup et al. 2005; Grieneisen et al. 2007). Box 3 presents the main features of different models developed to describe auxin transport and dynamics.

Box 3. **A brief introduction to auxin transport modeling**

Auxin movement involves active (through carriers) and passive (diffusion) transports. Auxin is actively transported through the cell membranes by exporters and importers, while its passive motion occurs in the cytoplasm, the apoplast, through the cell membranes (resulting mostly in import) and from cell-to-cell through plasmodesmata. Recently, transport through plasmodesmata has been estimated to be of a magnitude comparable to auxin active transport (Band 2021).

Auxin transport is usually modeled through ODEs that take the concentrations of the hormone auxin and transport carriers as variables (Kramer 2008; Rutten and ten Tusscher 2019). Some models explicitly consider the anionic and ionic forms of auxin, their different ratios at the apoplast and the cytoplasm, and their dependence on pH and membrane voltage (Band et al. 2014), while other models simplify auxin considering it a single variable (e.g. Grieneisen et al. 2007; Salvi et al. 2020).

Regarding transport, a basic component in all models is the active export of auxin through carriers and the polar positioning of these exporters. Yet, models can differ in the details of the pattern of polarity established (Grieneisen et al. 2007; Mironova et al. 2012; Tian et al. 2014; van den Berg et al. 2021). Models also differ in the inclusion of importers and their inhomogeneous spatial pattern of expression (Hong et al. 2017; Salvi et al. 2020). Diffusion across the cytoplasm and the apoplast and, more recently, cell-to-cell diffusion through plasmodesmata and apolar transporters (ABCBs) is explicitly modeled in grid-based spatial descriptions of the root, in which cells and the apoplast are described by several grid points (Mellor et al. 2020, 2022). Some models use vertex descriptions, where cells are single point entities and the cytoplasm and apoplast are not modeled explicitly and therefore cannot distinguish the contributions of each passive flux (Mironova et al. 2012; Hong et al. 2017).

While the polar distribution of exporters, as revealed by sophisticated imaging, is commonly established as an input and held constant, several models include that the concentration of carriers (exporters and/or importers) depends on auxin and other hormones and factors, although these dependences are distinct (Mironova et al. 2012; Salvi et al. 2020). Regarding the polarity of exporters, models have hypothesized that auxin fluxes polarize the exporters, showing that this feedback between polarization and auxin can drive self-organized patterns of auxin distribution (Mitchison 1980; Jönsson et al. 2006; Smith et al. 2006; Cieslak et al. 2015; Fàbregas et al. 2015; Marconi et al. 2021).

The models are established on semi-realistic root layouts, either through regular or irregular polygonal grids. The size and geometries of the cells and the level of realism used for cell geometries depends on the balance between simplification and the question being addressed. When cell division and cell elongation are included, the growth associated with these processes is modeled by adding new grid points. To avoid the sliding between cells of different layers, rules for radial coordination are imposed (Mähönen et al. 2014; Di Mambro et al. 2017; Salvi et al. 2020).

This type of modeling has been used to analyze the cross-talk between auxin and other hormones and to study the inhomogeneous distribution of carriers not only at the cell plasma membrane but also within the tonoplast (Moore et al. 2015; Di Mambro et al. 2017; Liu et al. 2017a; Salvi et al. 2020; Binenbaum et al. 2023).

A crucial aspect of auxin transport is that it is active, meaning that it involves energy consumption. This phytohormone uses exporters and importers to move from the cytoplasm to the apoplast in a process that requires energy. Active transport is very distinct from passive motion. While passive fluxes arise from concentration gradients and are directed to reduce them, active transport creates gradients, being active fluxes proportional to the local concentration of auxin. Importantly, exporters (PIN proteins) are localized anisotropically at the cell plasma membranes, driving polarity to auxin transport (Blilou et al. 2005; Wisniewska et al. 2006). For instance, in root vascular cells, exporters are located at the cell plasma membrane facing the root tip and not at the opposite cell membrane facing the shoot, driving a rootward auxin flux. In contrast, the polar positioning of exporters at the epidermis drives an opposed shootward flow. Modeling has been used to assess whether auxin polar transport, combined with auxin diffusive (nondirectional) movement, can drive the inhomogeneous distribution of auxin concentration and signaling that shows a maximum at the QC.

Through modeling, it has been shown that the fluxes arising from the measured polar distributions of PINs at the root tip result in a stationary, stable, and robust maximum of auxin concentration at the QC, even in the absence of QC-localized auxin synthesis (Grieneisen et al. 2007). Therefore, the polar distribution of exporters is sufficient to drive the auxin maximum. The polar lateral positioning of exporters at the meristem, facing inner tissues, drives a reflux from the shootward to the rootward flux, which strongly increases the auxin maximum (Grieneisen et al. 2007). Yet, the lateral expression of exporters is weak. In addition, local auxin biosynthesis occurring at the QC has been found (Ljung et al. 2005; Stepanova et al. 2008; Liu et al. 2017b), and its modeling has shown that it is sufficient to drive an auxin maximum at the QC when no reflux is present (Tian et al. 2014). Recently, the inclusion of passive auxin transport through plasmodesmata in the modeling of auxin dynamics (Box 3) has revealed that this transport promotes the reflux from the shootward to the rootward flux (Mellor et al. 2020; Band 2021), adding new mechanisms that can contribute to the reflux. Therefore, both the reflux and local biosynthesis may act redundantly to drive and sustain the auxin maximum at the QC, their relative importance being unknown (Brumos et al. 2018).

Initial studies used simple geometries and assumed a uniform capacity of all cells to import auxin, showing that further detail in these components is not necessary to account for the establishment and maintenance of the auxin maximum (Grieneisen et al. 2007; Mironova et al. 2010). The use of more direct reporters of auxin (Santuari et al. 2011; Vernoux et al. 2011) revealed additional inhomogeneities in auxin distribution, such as high concentrations in epidermal cells at the elongation zone and in the lateral root cap. Moreover, AUX1/LAX importers are distributed isotropically but not uniformly across the root (Swarup et al. 2001, 2005; Swarup et al. 2008; Péret et al. 2012). By including the spatial patterns of importers and more realistic cell morphologies in the modeling of auxin transport, the inhomogeneities of auxin distribution have been mimicked in silico (Band et al. 2014). This supports that the patterns of importers are relevant to concentrate auxin in specific layers, like the epidermis (Band et al. 2014).

Feedback pervades many aspects of the development of organisms, and auxin transport is no exception. Auxin regulates the concentrations of PIN proteins and the importer AUX1 (Blilou et al. 2005; Laskowski et al. 2006, 2008), which in turn regulate the flux of auxin. By modeling the feedback between PIN exporters and auxin, it has been shown that the position of the auxin maximum can emerge spontaneously, in a self-organized manner, without requiring detailed structural anatomy of the root, aside from a downward flow and a tip end (Mironova et al. 2010, 2012). Therefore, this feedback can be a crucial factor in driving an auxin maximum when the structural anatomy is disrupted, such as when the full polarity of exporters is not yet established, when the root tip is excised, or when cell death occurs in the columella (Mironova et al. 2010; Hong et al. 2017; Canher et al. 2020).

Since auxin upregulates the importer AUX1 (Laskowski et al. 2008), the feedback between AUX1 and auxin drives both local activation (a cell with high levels of auxin promotes its import of auxin, thereby increasing its auxin content) and lateral inhibition (the cell importing auxin depletes auxin from adjacent cells) (Smith and Bayer 2009). By modeling this feedback, we have learned that it can drive formation of periodic auxin maxima (Smith and Bayer 2009). Therefore, this feedback expands the well-known principle of local activation and lateral inhibition for the self-organized formation of patterns of chemicals (Meinhardt and Gierer 1974, 2000). We have also learned that it can prevent the simultaneous formation of auxin maxima on opposite sides of the primary root, thereby ensuring that only 1 lateral root is initiated at a time, either on the left or the right side (el-Showk et al. 2015). Moreover, it can explain the fragmented pattern of sieve element differentiation in the protophloem cell files found in specific mutants, which involves a pattern of auxin accumulation in some but not all cells (Moret et al. 2020).

While most analyses of auxin transport performed to date have focused on 2D longitudinal cross-sections, investigating the longitudinal auxin gradient, modeling of auxin transport across transverse (radial) cross-sections has also been performed to reproduce the radial pattern of auxin accumulation, with the highest concentration at the xylem axis (el-Showk et al. 2015). Moreover, 3D models of root segments have been developed when the interplay between longitudinal and transverse flows is considered a relevant factor in the process under study, such as in gravitropism and root hair development (Swarup et al. 2005; Jones et al. 2009). Since the analysis of plasmodesmata-mediated transport in longitudinal cross-sections supports the relevance of this transport for the relative weight of longitudinal vs transverse fluxes, the future inclusion of plasmodesmata in 3D models is expected to drive new insights.

### Root growth and zonation

In the stationary phase of root growth, cell division occurs only within a spatial region of the root, the meristematic zone. Cell elongation takes place in another confined spatial domain of the root (the elongation zone), just apically to the meristematic zone. Since auxin is an instructive signal for root growth by impinging on cell division and elongation, models that establish auxin transport dynamics along the root and drive an inhomogeneous distribution of the hormone have been extended to address whether auxin distribution can account for proper root zonation and growth.

In this regard, cell division and elongation have been included in models of auxin transport dynamics (Grieneisen et al. 2007; Mähönen et al. 2014; Di Mambro et al. 2017; Salvi et al. 2020). Given that the ultimate targets of auxin that regulate these cellular processes are unknown, hypothetical factors are included to direct these responses. While models have addressed zonation along the longitudinal axis, the mechanism behind the radial coordination of growth and zonation is unknown. Therefore, models impose this coordination and impede sliding between adjacent cells through hypothesized rules (Mähönen et al. 2014; Di Mambro et al. 2017; Salvi et al. 2020). The outputs of recent models, used for comparison with quantitative empirical data, include molecular gradients along the root and the number of meristematic cells at different times under wild-type conditions, as well as in altered scenarios where 1 or several molecular elements are modified (Di Mambro et al. 2017; Salvi et al. 2020).

These models have shown the relevance of another layer of complexity: the feedback between the cell dynamics and auxin signaling. For instance, PLETHORA TFs are long-lived molecules expressed in the root stem cell niche, are activated by auxin while repressed by cytokinin signaling, and act antagonistically to cytokinin signaling at promoting cell division and repressing cell elongation (Aida et al. 2004; Galinha et al. 2007; Mähönen et al. 2014; Salvi et al. 2020). The PLETHORA protein gradient extends more than its domain of transcription, and a combined modeling and experimental approach has shown that this gradient is formed by dilution through cell division and by cell-to-cell movement (Mähönen et al. 2014), these mechanisms also being present in other elongated tissues of animal embryos (Ibañes and Izpisúa Belmonte 2008). Therefore, cell-division-PLETHORA/auxin/cytokinin feedback is relevant to determine the number of meristematic cells (Di Mambro et al. 2017; Salvi et al. 2020).

The feedback between auxin signaling and cell dynamics has also been proposed to be crucial in determining the initiation of lateral roots (van den Berg et al. 2021). This initiation occurs periodically in time and is marked first by a transient increase in auxin signaling in the protoxylem at the transition zone to the elongation zone. Modeling has shown that anisotropy in cell shape, together with cell division and auxin reflux, is sufficient to spontaneously trigger oscillatory increases of auxin in these cells (van den Berg et al. 2021). On the one hand, anisotropic cell growth favors the accumulation of auxin inside the growing cell because of a more imbalanced ratio of import (occurring in all plasma membrane surfaces) to export (occurring only in the rootward, nongrowing, plasma membrane). This accumulation is higher in the longest cells of the vasculature, whose cells are narrower than those of other cell files. On the other hand, the oscillatory formation of auxin maxima is proposed to arise from out-of-phase meristematic cell divisions that cause periodic variation in the lengths of cells arriving at the elongation zone, resulting into variation in the capacity of cells to accumulate auxin.

Less attention has been paid to the transition to the mature zone, being the mechanism behind the cessation of cell elongation unknown. By modeling the cell dynamics in a 1D longitudinal layer along the elongation zone, it has been shown that the mechanism is not expected to rely on the duration of cell elongation, since it would be highly sensitive to differences in the proliferative capacity of the meristem and would result in a large variability in number of cells in the elongation zone (Pavelescu et al. 2018). Indeed, the variability observed in isogenic roots under the same conditions is constrained by the developmental mechanism at play. Therefore, by analyzing the pattern of variability, we can gain insights into the mechanism that drives it (Fig. 2, A to C). For instance, the number of cells in the elongation zone highly correlates with the ratio of meristematic production over cell elongation rates (Pavelescu et al. 2018). By modeling the 1D dynamics, it has been shown that this correlation is only mimicked in silico through a sizer mechanism, that is, if cells halt their elongation upon reaching a threshold cell size and not when reaching a threshold duration or spatial position (Pavelescu et al. 2018). Moreover, this correlation makes root growth proportional to the meristematic production rate, while disrupting this correlation would lead to highly variable root growth. It remains to be elucidated which mechanism enables cells to respond to their size by terminating their elongation. Cytokinin, through auxin, promotes cessation of cell elongation, potentially by stiffening the cell walls (Liu et al. 2022). Accordingly, it may be proposed that cell wall stiffness increases with cell size, and cells terminate their elongation when becoming too stiff, effectively conducting a sizer mechanism.

### Root growth as a mechanical process

From a physical perspective, root growth is a self-organizing, out-of-equilibrium process that uses and dissipates energy. Forces and stresses vary and are spatiotemporally regulated by the plant, a process that requires energy (Coen and Cosgrove 2023). Energy consumption by cells enables them to constantly generate mechanical perturbations, namely cell wall loosening, which drives the influx of water and thus enlargement of the cell (Cosgrove 2016, 2024). Recently, several modeling efforts that establish mechanical descriptions of the root have been formulated.

**Figure 3.**
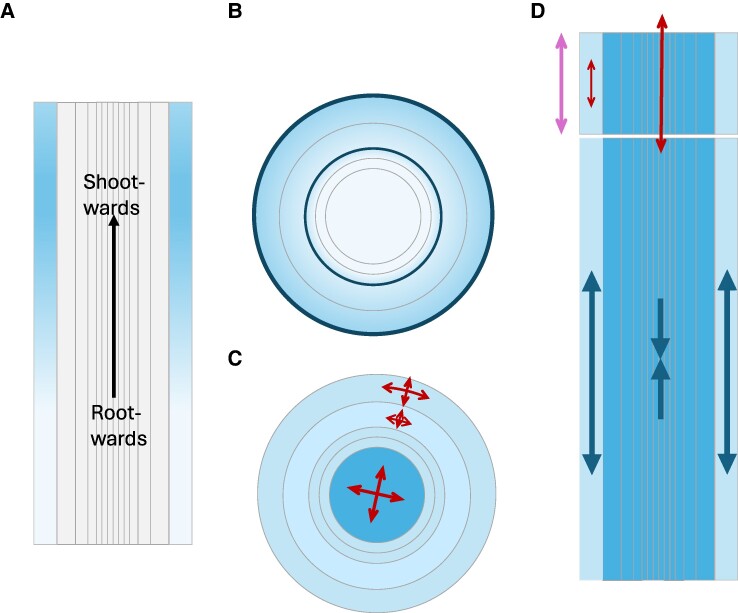
Mechanical aspects of root growth. Cartoons of the anisotropy and gradients of mechanical variables such as stiffness **(A, B)** and cell wall loosening **(C, D)** in longitudinal (A, D) and transverse cross-sections (B, C). In the case of D, the results correspond to hypocotyl elongation. Models of tissue mechanics have characterized these magnitudes through comparison with true anatomies under perturbations. A) There is a gradient of longitudinal cell wall stiffness in the epidermis (outer layer) along the axis of elongation, with cells located shootward showing greater stiffness (darker blue) than those positioned rootward (lighter blue) (Liu et al. 2022). B) Radial cross-sections of root growth show a dual-ring of transverse stiffness, the epidermis and endodermis being the tissues with the greatest stiffness (darker blue) (Fridman et al. 2021). C) Radial cross-sections show non-uniform extensibility (red arrows and blue shade), with internal tissues being more extensible than outer layers (Fridman et al. 2021). D) The epidermis in the hypocotyl has lower extensibility than inner tissues (red arrows, blue shade), partially due to the wider walls of its cells, while the degree of elongation of all tissues is the same (purple arrow) (Kelly-Bellow et al. 2023). This drives conflicting growth that results in tensile stress in the epidermis and compressive stress in inner tissues (blue arrows) (Kelly-Bellow et al. 2023). It remains to be seen whether the root has a similar or distinct pattern of longitudinal extensibility and stress.

One aspect addressed is whether the stiffness of cell walls in root cells is isotropic (Liu et al. 2022). The stiffness of a material determines how much it elongates when subjected to a force; the higher the stiffness, the smaller the elongation. Plant cells are subjected to turgor pressure, and their volume depends on this pressure and the cell wall stiffness. Are root cell walls equally stiff longitudinally (i.e. in the direction of root elongation) than in transverse directions? Since the stiffness can be studied by evaluating the elongation in response to a force (Majda et al. 2022), a 3D root segment (from the tip to the elongation zone) has been modeled as composed of epidermal cells whose walls are a hyperelastic material with fibers, and its mechanical relaxation in response to an increase in turgor pressure has been simulated (Liu et al. 2022). To simplify the 3D analysis and focus on the epidermis, the inner tissue has been modeled as a single homogeneous material. Because relaxation is a very rapid process compared with the slow dynamics of root elongation, the model did not include cell division nor cell wall loosening. Moreover, by assuming that this relaxation is a reversible process, it has been compared with measurements of the shrinkage of epidermal cells at the root tip when living roots are grown in hyperosmotic solutions. This comparison has been possible because the model used initial realistic cell sizes. The combined modeling and experimental approach has shown that only those simulations with anisotropic epidermal cell wall stiffness, with cell walls being softer in the longitudinal direction than in the transverse direction, matched the measurements (Liu et al. 2022) (Fig. 3, A and B). Therefore, these results support the idea that epidermal root cell walls are softer in the direction of their elongation.

Additionally, the measurements in living roots have shown that the shrinkage is not uniform along the longitudinal axis, being smaller for larger cells and being regulated by cytokinin hormone (Liu et al. 2022). The model discarded that the distribution of cell lengths along the root could result into this non-uniform shrinkage. Accordingly, it has been proposed that cells increase the longitudinal stiffness of their walls as they elongate, thereby facilitating their growth arrest (Liu et al. 2022) (Fig. 3A). Since this increase in stiffness has not been modeled yet, future challenges in modeling include mechanically simulating the arrest of cell elongation through a gradient of cell wall stiffness.

Another aspect addressed is whether root growth involves different mechanical properties among concentrical cell layers. To that end, the 2D cellular and the cell layer structure of the root in a transverse plane has been modeled (Fridman et al. 2021). Initial realistic cell shapes were considered to compare the morphology of the time-evolved simulated root to data from transverse sections of real roots. Since the process to be modeled was irreversible root growth, the mechanical description of cells included cell division and cell wall loosening, besides cell wall stiffness. To model the irreversible process of cell wall loosening, the resting length of cell walls (or its extensibility) is imposed to increase over time (Lockhart 1965; Bassel et al. 2014; Boudon et al. 2015; Fridman et al. 2021). The cell wall resting length corresponds to the extension it would be when in isolation and in the absence of forces. Note that the actual length of cell walls is not the resting length but rather the one resulting from all internal (and external, if present) forces at play.

This approach has been implemented through mass-spring models (MorphoDynamX) to simulate the transversal growth of roots at the meristematic region (Fridman et al. 2021). The model results have been compared with morphological data on the wild type and mutants of brassinosteroid signaling. Through this comparison, it has been proposed that transversal cell wall loosening, and stiffness differ between inner and outer tissues, the epidermis and endodermis being stiffer tissues (Fridman et al. 2021). Therefore, distinct root cell layers exhibit different mechanical properties in the transversal plane (Fig. 3, B and C).

It remains to be elucidated whether inner and outer cell layers also exhibit different mechanical properties along the longitudinal direction. Based on the results of hypocotyl elongation (Kelly-Bellow et al. 2023), we may expect it is the case. The elongation of a segment of hypocotyl has been studied with a similar approach to the previous one but implemented through continuous elastic theory (MorphoMechanX), which facilitates calculation of the stresses (Kelly-Bellow et al. 2023). The results support that the outer epidermal layer of the hypocotyl has less cell wall loosening than the inner tissue (Kelly-Bellow et al. 2023). The epidermis is under tension (being pushed by the inner tissue to grow more), while the inner tissue is under compression (being pulled by the epidermis to grow less) (Fig. 3D). Moreover, brassinosteroids reduce these stresses by enhancing the loosening of the epidermis (Kelly-Bellow et al. 2023). The relevance of brassinosteroids in the epidermis for ensuring proper root growth (Savaldi-Goldstein et al. 2007; Hacham et al. 2011) suggests that a similar pattern of stress and differential loosening can be expected in the root itself.

The previous models do not address which mechanism drives changes in cell wall stiffness and cell wall loosening. Since, the acid growth theory proposes that auxin lowers the pH of the apoplast, thereby enabling cell wall loosening (Rayle and Cleland 1970), a model to investigate the relationship between auxin and root elongation mechanics has been formulated (Marconi et al. 2021). The model focused on the first appearance of anisotropy and its early maintenance, modeling the dynamics from the embryo heart stage until initiation of root elongation. It assumed that cell wall stiffness decreases for intermediate concentrations of auxin and cell wall loosening was not modeled since the elongation and mature zones of the root were not included. To reduce the computational cost, a 2D polygonal description of cells was made and implemented through position-based dynamics (Müller et al. 2007). The results have shown that self-organized elongation of the root can arise when a few initial asymmetries and feedback between auxin, polarity, and stiffness are assumed to take place. In the model, cell wall stiffness is reduced at intermediate auxin concentrations, and the auxin concentration and the cell growth anisotropy regulate the polar localization of PIN proteins. This feedback reinforces anisotropic cell and tissue elongation once it has started. Among other questions, the roles of an increase in cell wall stiffness to transition to the mature zone and of cell wall loosening through irreversible extension (and not stiffness) remain to be addressed.

While the previous approaches model individual cells within segments of the root, coarse-grained descriptions of the whole primary root have been also formulated (Porat et al. 2024a, 2024b). In coarse-grained descriptions (Kramer 2008), the root is modeled as a continuous material since many cells are present. These descriptions have been used to evaluate the whole shape of the primary root when it grows under an environmental stimulus like gravity (Porat et al. 2024b). Roots grow straight when growing on a vertical plane and coil when growing on a horizontal surface. The results suggest that these root shapes can be explained by the action of gravitropism and proprioception and that additional tropisms involved, like the response to touch, do not contribute significantly (Porat et al. 2024b).

## Concluding remarks

The development of organisms is a highly regulated self-organizing process, such that the organism’s functioning and shapes are repeatedly and robustly generated for each organism from a single cell. By examining specific patterning and morphogenetic processes through modeling, we seek to gain insights into the principles of self-organization. Yet, different mechanisms and models can drive the same shape and pattern (Boudon et al. 2015). To identify the actual mechanism or mechanisms at play, it is essential to evaluate how the shape or pattern depends on the elements that generate it by making model-specific predictions. One approach is to compare the model predictions with quantitative data. The detailed 3D and 4D anatomical characterizations of the root meristem (Fridman et al. 2021; Graeff et al. 2021) provide extensive data for future comparisons of results on root growth from models with actual root anatomy at the single-cell level. Consequently, the number of cells in each zone, as well as their shapes along the longitudinal axes, can now be used to test models more precisely and discard mechanisms of root growth, whether these models focus on hormonal signaling regulation of cell dynamics or on mechanical interactions. Another approach is to assess the dynamics of formation, as dynamics contain much more information than the stationary state and can thus test the validity of models. While the pattern or shape achieved by different models may be the same, the dynamics are not and can be used to discard models. Advancements in the visualization of gene expression and proteins are now allowing us to reveal their dynamics, enabling the testing of existing models and the development of new ones (Winter et al. 2024).

Many challenges lie ahead. The quantification of gene expression has shown that it is highly dynamic (Shahan et al. 2022). Moreover, by assessing dynamics at the molecular level, the presence and relevance of fluctuations will be unveiled. Among the many possibilities that stochastic molecular fluctuations offer is the ability to enable stable cell fate choices through heterodimer formation (Lord et al. 2019). While many heterodimers occur in the root stem cell niche, the effect of stochastic fluctuations in heterodimer dynamics and whether these can facilitate cell fate decisions have not been investigated yet.

Dynamics in signaling are also relevant. An increasing body of evidence indicates that hormone signaling can direct fast and slow responses. The different time scales of these responses, when coupled to other processes which have their own time scales (like cell division and cell elongation), are relevant, and modeling can be very helpful in evaluating their impact. That said, the modeling of both fast and slow signaling dynamics is still in its infancy (Mähönen et al. 2014; Großeholz et al. 2022). In addition, dynamics are also relevant for the information encoded in signaling or the response mechanisms, which are rarely understood. This is particularly true for auxin signaling, leading models to commonly make assumptions about what is encoded. Is the response to auxin solely based on its current concentration in the cytoplasm, or is the response based on a temporal integration of auxin concentration? Or do cells detect fold changes (Adler and Alon 2018) in auxin concentration? Alternatively, are spatial gradients of auxin hormone encoded such that the cell responds to them? In this regard, it has recently been demonstrated that cell response to auxin is consistent with the integration of auxin signaling over time as opposed to a response to current levels (Galvan-Ampudia et al. 2020; Santos Teixeira et al. 2022). As modeling has shown in the shoot apical meristem of Arabidopsis, temporal integration smoothens fluctuations, leading to robust responses (Hong et al. 2016). Temporal and spatial integration of stimuli is a characteristic of bacterial chemotaxis that enables robust detection (Rode et al. 2024), and plant roots may use similar strategies in some contexts.

Given that root growth is one of many examples of tissue elongation that occurs in living organisms, it is expected to share common physical principles with other similar processes (Boutillon et al. 2024). A conserved mechanism proposed for tissue elongation is based on intrinsic growth that is inhomogeneous, acting only at the root tip and on the anisotropy of material properties, which are more fluid-like when matter is added and become more rigid afterwards (Boutillon et al. 2024). In the root, growth is inhomogeneous since it happens only in the basal part of the root (i.e. the meristematic and elongation zones). It is also anisotropic since most cell divisions take place in planes transverse to the longitudinal axis and cell elongation occurs mostly longitudinally. In addition, the material properties of the root are anisotropic (Gorelova et al. 2021; Coen and Cosgrove 2023). While the proposed conserved mechanism explains the elongation of cells with cell walls, such as pollen tubes (Boutillon et al. 2024), the physical mechanisms driving root elongation remain to be elucidated. The use of physical models that simulate root growth and comparison of these observations with new quantitative data measuring relevant physical variables will be essential for answering these questions.

**Outstanding Questions**

- Which are the temporal dynamics of gene expression and proteins within cells at the root, and how do these dynamics control root development and growth?
- Which are the properties that the short-range movement of transcription factors between cells confer at the root stem cell niche?
- Which are the slow and fast dynamics of hormonal signaling, and how do root cells respond to them?
- Which is the mechanism that enables root cells to respond to their size by terminating their elongation?
- Which are the physical mechanisms driving root elongation?
- How are these physical mechanisms related to environmental stimuli and hormone signaling?

## Data Availability

The data underlying this article will be shared on reasonable request to the corresponding author.

## Dive Curated Terms

The following phenotypic, genotypic, and functional terms are of significance to the work described in this paper:

WOX5 Gramene: AT3G11260
WOX5 Araport: AT3G11260

BES1 Gramene: AT1G19350
BES1 Araport: AT1G19350

auxin CHEBI: CHEBI:22676

initial AmiGo: PO:0004011

root AmiGo: PO:0009005

AUX1 Gramene: Os01g0856500
AUX1 Araport: Os01g0856500
